# The Influence of Dentists' Profile and Health Work Management in the Performance of Brazilian Dental Teams

**DOI:** 10.1155/2021/8843928

**Published:** 2021-11-03

**Authors:** Suellen da Rocha Mendes, Renata de Castro Martins, Juliana Vaz de Melo Mambrini, Antônio Thomaz Gonzaga Matta-Machado, Grazielle Christine Mattos-Savage, Jennifer Elizabeth Gallagher, Mauro Henrique Nogueira Guimaraes de Abreu

**Affiliations:** ^1^Graduate Program in Dentistry, Faculty of Dentistry, Federal University of Minas Gerais, Belo Horizonte, Minas Gerais, Brazil; ^2^Department of Community and Preventive Dentistry, Faculty of Dentistry, Federal University of Minas Gerais, Belo Horizonte, Minas Gerais, Brazil; ^3^René Rachou Institute, FIOCRUZ, Belo Horizonte, Minas Gerais, Brazil; ^4^Department of Preventive and Social Medicine, Faculty of Medicine, Federal University of Minas Gerais, Belo Horizonte, Minas Gerais, Brazil; ^5^Centre for Host Microbiome Interactions, Faculty of Dentistry, Oral & Craniofacial Sciences, Kings College London, London, UK

## Abstract

To evaluate the association between dentists' profile and health work management with the performance of primary care dental teams in the Brazilian National Health System, both nationally and regionally. Secondary data analysis from a Brazilian National Programme that evaluated 18,114 Brazilian dental teams, working in the public sector, between 2013 and 2014. Twenty-four independent variables taken from dentists' profile and dental team management characteristics were analysed to assess their influence on reported “dental team performance.” An estimated score was generated from their performance on 20 dental procedures by an item response theory model. Multiple linear regression models were performed for each Brazilian geographical region, separately and for the whole of Brazil. *p* values ≤ 0.05 were considered significant. Two variables related to dentists' profile, “having graduate studies” (*β* = 0.151) and “undertaking continuing professional development training” (*β* = 0.101), were associated with enhanced dental team performance in all five Brazilian geographical regions and nationally. The dental team management variables of “having a flexible dental appointment list” (*β* = 0.218) and “monitoring oral health indicators” (*β* = 0.132) also contributed to improve team performance in each of the regions and nationally. Dentists' profile influenced the performance of dental teams from south region more than the other regions. The findings suggest that continuing professional development, including postgraduate education, and strategic management characteristics are important for primary dental care performance and should be reflected in health policy initiatives in support of quality care. Regional factors could be considered for health care management.

## 1. Introduction

Primary health care (PHC) is the most important level of care of the Brazilian National Health System (known as Sistema Único de Saúde—“SUS”), where all the citizens can have access to health promotion, prevention, and treatment, through integrated services and free of charge at the point of delivery. The National Oral Health Policy (NOHP), published in 2004, defines the inclusion and organisation of the dental teams at the SUS, comprising strategies to expand and improve the health care provided to the population [1–3].

The importance of continuing professional development training and having an adequate dental workforce to meet local demands is outlined in the NOHP, as the epidemiological and territorial analysis to the proper planning of the oral health care provision. This adequacy is essential at SUS, due the great sociodemographic differences observed across the Brazilian Geographical Regions [4]. Also, having proper working conditions, with reasonable dental facilities and complete supply of materials and instruments, is essential for teams to work effectively [1, 3].

Evidence suggests that although structural and organisational factors may influence the health care provision, factors related to the workforce, such as professional development training and labour aspects as salary, work shifts, and employment bond, may also influence it [5, 6]. In the last decades, the growth of work precariousness, informal employment bond, and salary deterioration resulting from a neoliberal logic of employment can also be observed in the SUS ways of employment [7, 8]. Together, these are possible influences on health care delivery.

With the aim of improving access and quality of services provided by the SUS, the Brazilian Ministry of Health (MofH) financed, between 2011 and 2018, the “National Programme for Improving the Access and Quality of Primary Care—PMAQ-AB.” Compliance with the programme was voluntary, and those teams that demonstrated good outcomes in the evaluation process received a financial incentive. Concerning the dental care services' evaluation, it included information on the primary health care unit/dental facilities structure, availability of dental instruments, dental procedures executed, dentists' profile, and service organisation [9].

Preliminary analysis of data from the second evaluation cycle of PMAQ-AB, occurred between 2013 and 2014, showed that it is necessary to expand the provision of dentures and dental prostheses [10]. The psychometric analyses of the questions related to the execution of the dental procedures attributed a score to the dental teams that we called “dental team performance,” varying between -3.66 and +1.87 (mean of -0.06; median of +0.01) and showed that these items were better in discriminating the dental teams with negative scores, especially those between -2 and -3 [11]. These preliminary analyses were necessary to achieve the aim of the present study, that is, to evaluate the influence of dentists' profile and health work management in the performance of primary care dental teams of the Brazilian National Health System. First, at national level, and second, by Brazilian Geographical Region. The null hypothesis is that dentists' profile and work management do not affect the performance of primary care dental teams in the national and regional level.

Although preliminary studies have already assessed the same database for evaluating the Brazilian health system [12, 13], this is the first to propose a methodology for estimating the dependent variable from a psychometric analysis of the items, in addition to assess the influence of dentists' characteristics and organization of health services on this outcome.

Considering the SUS has recently completed 30 years, the NOHP is only 16 years old [1, 3]; ongoing evaluation and adjustments are important and necessary. Evaluating if, and how, the dentists' profile can influence the performance of Brazilian dental teams working at SUS can help the improvement of the services provided to the population. Identifying aspects related to continuing professional development, employment bond, and career plans that are important for the good performance of such teams, greater incentive can be given to complementary training and the provision of internal training at the SUS. In addition, new public policies that guarantee greater employment stability can be considered.

## 2. Methods

### 2.1. Ethics Statement

This study was approved by the National Ethics Research Council and by the Research Ethics Committee of the Federal University of Minas Gerais (Protocol No. 02396512.8.0000.5149; Approval No. 2004382) to gain access to the Brazilian Ministry of Health database.

### 2.2. Study Design

This is a secondary analysis of data from the second evaluation cycle of PMAQ-AB, a Brazilian public sector evaluation of primary health care services, between 2013 and 2014. Of the 23,150 dental teams working at SUS in 2013, a total of 18,114 (78.2%) dental teams were evaluated regarding access to dental care and quality of services provided to the Brazilian population [9].

### 2.3. Data Collection and Variables

All the questions included in this study were obtained from the external evaluation phase of PMAQ-AB programme, consisting of face-to-face interviews with a dental representative of each PHC unit. The issues were based on the PHC principles and on the Donabedian's evaluation model for health services, based on structure, process, and outcomes [14]. All the interviews were conducted by a trained professional, following 40 hours of training and evaluation regarding PMAQ-AB criteria.

In our analysis, the dependent variable was “dental team performance” obtained through dentist's report about the execution of 20 mandatory dental procedures of PHC dentistry and presented in full in our previous paper [11]. The questions included the execution of common dental procedures (preventive, restorative/prosthetic, surgical, and endodontic), besides the identification, referral, and monitoring of oral cancer cases.

These 20 procedures were evaluated by the item response theory (IRT), a mathematical model that relates the probability of an individual's response to an item and its latent trait [15]. The latent trait of this study was “dental team performance,” identified as a score that could range from -4 to +4. Through the IRT, items' psychometric characteristics were estimated, and a one-off performance score was attributed to each dental team evaluated in the second cycle of PMAQ-AB. In the present study, dental team scores ranged from -3.66 to +1.87 (mean of -0.06; median of +0.01) [11, 15].

The independent variables were organised into two categories, with 13 questions concerning the dentist profile (human resources factors) and 11 questions regarding the health work management of dental teams (Table 1). The questions included were mostly categorical (22 questions) and were dichotomized (yes/no) for the statistical analysis. The variable “continuity professional development training” was a quantitative variable that ranged from 0 to 8, according to the number of activities in which the dentist participated; the variable “covered population” could vary from 1 to 9, according to the number of PHC teams the dental team assisted at the time (one PHC team cover about 3,500 patients).

### 2.4. Statistical Analysis

The items included in the dependent variable estimation—"dental team performance”—were descriptively analysed in a previous paper by Mendes et al. [10]. For the independent variables, univariate analysis was conducted to estimate frequencies according to the absolute and relative values. Bivariate analysis used linear regression. Initially, each of the 24 independent variables was included in the simple linear regression model with the dependent variable, “dental team performance” scores, estimated by IRT. Those variables with *p* values ≤ 0.20 were included together in the multiple linear model. Only those variables that showed statistical significance (*p* ≤ 0.05) were included in the adjusted model. Collinearity was tested by the “variance inflation factor—VIF,” and values below the value of two were considered acceptable.

Given the great differences between the Brazilian geographical regions regarding population socioeconomic status and access to dental care, the multiple linear regression model was also run by region—north, northeast, centre-west, southeast, and south. The beta value and its confidence intervals are presented to those variables that showed statistical significance (*p* ≤ 0.05) in the final model, according to each region.

To evaluate the residues, the standardized and studentized deleted residuals were obtained as a histogram and normal probability plot to evaluate its theoretical assumptions. The homoscedasticity assumption was verified, and the normality distribution of residuals checked by the observation of the histogram. The final model summary of each linear regression model was estimated to verify how much the independent variables could explain the variation of the dental team performance.

Data were organised, dichotomized, and analysed in the IBM SPSS Statistics software, version 25 (IBM Corp. Released 2017. IBM SPSS Statistics for Windows, Version 25.0. Armonk, NY: IBM Corp.).

## 3. Results

Data from the second evaluation cycle of the national dental system (PMAQ-AB, 2013-2014), relating to access and quality of SUS primary care dentistry, involving the questionnaire responses of 18,114 dental teams were analysed in the present study. No missing data were observed amongst participating teams.

Table 2 provides a descriptive analysis of each categorical independent variable included in the present study, at national and regional level. Overall, there was great variation between the five Brazilian geographical regions (Table 2). Dentists' profile (human resources factors) differed by region, with those working in the south region more commonly reporting complementary training and involvement in academic activities. The south region also showed better labour relationships (having been hired by public tender and being employed as a civil servant), career plans, and financial incentives when compared with the national profile, whilst those in the northeast showed lower frequencies of these variables. In regard to the “health work management” variables, the southeast had higher frequencies of monitoring and planning of activities and easier access to appointment scheduling and referral to secondary care, comparing with Brazilian frequencies, while the north had lower frequencies of these variables.

Table 3 provides an overview of the quantitative independent variables “continuing professional development training” and “number of PHC Teams that the dental team assist.” On average, dental teams in the southeast and south regions performed more continuing professional development training and the north less, compared with the national average. Dental teams from southeast and south regions were, on average, responsible for a greater population coverage, compared with other geographical regions and nationally.

Simple linear regression and adjusted model were first performed for Brazil as a whole, using the performance of the dental team as dependent variable and the above-mentioned items as independent variables. Independent variables showing *p* values ≤ 0.20 in the simple linear regression model were included in the multiple regression model using the “Stepwise Forward Method.” The variables “master's degree/doctoral studies,” “dentist's employer,” and “career plan with progression for titles and professional improvement” were excluded from the model by this automatic method.

In this preliminary multiple model, analysis of collinearity diagnosis indicated that variables “employment bond” and “career plan” had high variance inflation factor values (VIF = 3.168 and 5.265, respectively) and should be removed. After manual adjustment of the model, the collinearity of the remaining variables showed VIF values lower than 2, which was acceptable.

Thus, Table 4 presents the final adjusted model for Brazil as a whole, where only the variables that showed statistical significance are presented. It is possible to observe that having graduate studies, performing continuing professional development training, tutoring undergraduate students, having been hired by public tender test, and having a career plan with progression for time of service, with progression for professional performance and financial reward for performance were associated with the enhancement of the dental team performance. Considering the variables related to work management, all the mentioned variables had a positive impact in the performance of the dental team.

Table 5 illustrates the findings for each Brazilian region, including only the beta values and confidence intervals of variables that remained in the adjusted models. Two were present across all five regions: “graduate studies” and “continuing professional development training.” The results suggest that variables related to the dentists' profile influenced the performance of dental teams from south region more than the other regions, especially having “graduate studies,” “career plan with progression for time of services,” and “financial incentive for performance.” On the other hand, dental teams from the north were influenced only by “graduate studies,” “continuing professional development training,” and “employment bond (civil servant).”

The southeast showed the highest number of work management variables influencing dental team performance. The variables “monitoring and analysis of health care indicators” and “flexible dental appointment list” also influenced the performance of the dental teams in all the five regions.

The histogram and normal probability plot showed a normal distribution of the residuals. The model summaries suggest that the independent variables included in the analyses could explain about 22% the variation of the dental team performance. Concerning the variation by geographical region, the adjusted *R*^2^ shows that such values are representative.

## 4. Discussion

In the present study, having “graduate studies” (i.e., postgraduate education/qualification) and undertaking continuing professional development training were positively associated with dental team performance, nationally, and in each of the five regions of Brazil. Regional differences were observed regarding the influencing variables, with the north being influenced by a lower number of variables relating to dentists' profile, when compared with other regions and Brazil overall suggesting inequalities in care provision.

A similar finding was observed by Cunha et al. [12], evaluating the same dataset where they observed that dentists performing continuing professional development provided more dental prostheses in the PHC [12]. In the same direction, a study from Baumgarten et al. [13] had identified that management and dentist's profile factors were associated to the performance of five primary care activities, such as restorations, tooth extraction, scaling and planning, and pulpectomy [13]. Despite our findings confirmed other studies using the same dataset, both studies [12, 13] had evaluated few dental procedures, with no evaluation of its psychometric properties. Given the importance of the dental workforce in serving the Brazilian population through SUS primary care dentistry, and the importance of monitoring and planning the health services to suit their needs in health care, the present study provides relevant information, especially for evaluating how these aspects influenced the performance of the dental teams. Dentists should receive adequate education and continued training to be prepared for the types of care needing to be delivered for the population [6].

The Brazilian National Health System—SUS—has recently had its 30^th^ anniversary; however, global WHO policies [16] together with the UK National Health Services (NHS) celebrating over seven decades lend support to the view that primary health care (PHC) is the most appropriate, safe, cost-effective, and sustainable way of organising health systems [17, 18]. PHC is delivered by SUS through the Family Health Strategy Programme (FHS), which is the gateway to the health system and an important tool to promote social justice and health, especially to the low-income population. The FHS is structured to solve 80% of population's health demands, in addition to coordinating a network of care, together with being a point of reference and counterreference for specialized care [2].

In Brazil, the main organisational strategy for the qualification of SUS workforce is the National Policy of Permanent Health Education that seeks to break with a curative biomedical model, which is contrary to the essence of the Brazilian Health System [19]. Santos and Hugo (2018) reported that dentists having specialisation training in Family Health resulted in greater activity in line with the proposals and principles of the SUS primary care, such as offering oral health promotion activities and conducting home visits [20].

Although the current study included not only the Family Health specialisation training, but all types of specialisation trainings in dentistry, it corroborates findings which suggest that specialist training can assist with the essential principles and the mandatory dental procedures of SUS [20]. It is quite important this type of training to provide dentists with greater knowledge and, thus, better care for patients. It is worth mentioning that such training should increase the quality of oral health care and not restrict the primary care to a single dental specialty [6, 8].

Despite specialization courses being restricted to professionals who pay for their additional training, the SUS provides to its workforce access to a variety of continuing professional activities. It can be highlighted the matrix support and the telehealth, a specialised technical support for PHC teams that allows the integration of PHC with the health system by boosting communication between teams, contributing to increase the resolution capacity in PHC. Based on interdisciplinary discussion and knowledge exchange, it seeks the multidisciplinary discussion of complex patients and clarification of doubts related to treatments [19, 20].

In a study proposing actions to strengthen PHC in the SUS, Tasca et al. [17] emphasize the need of planning supply of human resources, developing a professional training plan with emphasis on SUS specificities, and having a permanent and sustainable strategy for providing PHC professionals in needed areas [17]. This not only reinforces the importance of professional qualifications to suit the local care model and the needs of the current and future designated population but also suggests that the training designed for the SUS workforce indeed is efficient and should be encouraged [20]. Health policy and funding initiatives should support professional career development in support of quality care.

This research also showed that all the variables related to planning and organisation of dental care services by local demand influenced dental team performance. The variables “monitoring and analysis of population's oral health indicators by the dental team” and “flexible dental appointment list,” had a positive impact in all the five regions, by assisting scheduled and walk-in patients. Our findings reinforce the importance of the evaluation and local planning of services, improving and adapting different assessment methods [21].

Even though scheduled appointments represent better organisation of health care services, access by walk-in patients to healthcare suggests that different demands in society are being met, especially considering urgent cases of pain. This is important to address social inequity given the research evidence that people from lower socioeconomic groups are more likely to access care on an emergency basis [22]. On the other hand, high demand for urgent dental care can also be a consequence of long waiting times for dental appointments or incompatible opening times of health services for full time workers [22, 23]. Referrals to secondary care may indicate good care pathways within the health care network. In fact, the SUS network model, in which primary care coordinates the flow, is considered more effective in terms of internal organisation, resource allocation, and clinical management, which may influence the performance of dental teams [24].

There were different variables influencing the dental team performance according to each Brazilian geographical region. This can reflect their differences in dental care needs, which highlight the importance of understanding each region to ensure equity in dental care delivery. Still, these findings can also suggest that some dentists are not able to perform all dental procedures required by the local demand and what further training and support should be offered to them [10].

The independent variables included in this study explained only about 22% of the dental team performance variation. Nonetheless, in social sciences, it is a relevant finding; however, to understand a complex health care system and what can influence in the performance of the teams working on it, it is important to evaluate other aspects besides dentists' profile and work management. Also, the performance of dental teams was estimated from the execution of 20 mandatory dental procedures of SUS primary care and could vary if other variables were considered. The cross-sectional design of this survey does not allow to achieve causal relationships, but rather associations. Thus, longer term monitoring is recommended.

Although the present study analysed secondary data from the second evaluation cycle of PMAQ-AB, in which municipal managers selected participating dental teams, and the answers were dentists' report about the dental teams work process, the program included almost 80% of the dental teams working at SUS in 2013-2014 and covered all the five Brazilian geographical regions, which represents a relevant dataset from the dental teams working at SUS primary care in dentistry.

Our results suggest that internal continuing professional development activities must be maintained and even expanded, aiming to support dental teams in planning and execution of individual and collective care. The planning of care services through epidemiological assessments and the organisation according to local demand must be constant in the primary care of SUS, aiming to provide assistance for all who need it—scheduled and walk-in patients—and according to local needs, articulating the primary care dentistry with other specialities and with the other levels of care. Inequalities in the different Brazilian regions should be also taken into account in planning health care.

It is important to note that characteristics that influenced dental teams in some Brazilian geographical regions, specifically, should also be observed, although in the present study, they have not been discussed in depth, such as employment bond and career plan. In studies conducted in developed countries, characteristics related to recruitment, employment, hiring, deployment, migration, health safety, and retirement proved to be relevant both in the work process of professionals and also in population's health outcomes and should be the focus on future studies [25]. In addition, other variables that are not limited to dentists' characteristics and the organisation of health services in the SUS should be evaluated in future studies, aiming a better understanding of the Brazilian health system and the implications for the performance of primary care dental teams.

The findings suggest that quality of primary dental care is associated with the profile of the lead dentist, most notably those related to further education progress and professional training. In addition, dental teams' work management is important in different domains, specially concerning to the monitoring of population's oral health and a flexible dental appointment list. This study showed variations between regions and the importance of encouraging dentists to perform continuing professional development courses and graduate studies, especially those that can improve the capacity to assist the SUS, aiming the improvement of the services delivered to the population. Also, it reinforces the importance of proper evaluation and planning of dental services to reach the local demand along the country and highlights that further research is required to examine at other aspects that could influence dentists' and dental team performance in the SUS year by year, ensuring that the findings influence future action.

## Figures and Tables

**Table 1:** Questions of the independent variables, related to dentists' profile and health work management of the dental teams, and the possible answers. Brazil, 2013-2014.

Dentists' profile
1. Does the dentist have graduate studies? Yes; No.
2. Does the dentist have master's degree and/or doctoral studies? Yes; No.
3. Does the dentist tutor undergraduate students? Yes; No.
4. What was the hiring process of the dentist? Public tender test; Other.
5. Which employment bond does the dentist have? Civil servant; Other.
6. Who is the dentist's employer? Local government direct contract; Other.
7. How long have the dentist been in the dental team? Two years or less; More than three years.
8. Does the dentist have career plan? Yes; No/do not know.
9. Does the career plan have progression for time of service? Yes; No/do not know.
10. Does the career plan have progression for professional performance? Yes; No/do not know.
11. Does the career plan have progression for titles and professional improvement? Yes; No/do not know.
12. Does the dentist receive financial incentive for good performance? Yes; No.
13. Does the dentist perform continuing professional development training? Score ranging from 0 to 8 according to the number of the listed activities performed.

Health work management of the dental team
1. Is the PHC unit a trainee field for undergraduate and graduate students? Yes; No.
2. Does the dental team have documents proving the monthly planning and scheduling of activities? Yes; No.
3. Does the dental team monitor and analyse population's oral health indicators? Yes; No.
4. Does the dental team receive support to plan and organise the labour process? Yes; No.
5. Does the dental team have access to data to analyse population's oral health status? Yes; No.
6. Does the dental team have documents proving self-evaluation in the past six months? Yes; No.
7. Does the dental team take part in the PHC team meetings? Always; Sometimes or never.
8. How is organised the dental appointment list? Flexible dental appointment list (scheduled and walk in patients); Restricted dental appointment list (scheduled patients only or walk in patients only).
9. When are the dental appointments scheduled? At any day and any time; At fixed days and hours.
10. Can the dental team refer the patients for secondary care? Yes; No.
11. How many PHC teams the dental team assist? (one PHC team cover about 3,500 patients) Score ranging from 1 to 9 according to the number of PHC teams.

Questions obtained from the external evaluation phase of PMAQ-AB programme, for the face-to-face interviews with the dental representative. PHC: primary health care.

**Table 2:** Frequency of the categorical independent variables related to dentists' profile and work management of PHC dental teams, Brazil, and according to each Brazilian Geographical Region, 2013-2014.

Dentists profile and management items	Brazil (%) *N* = 18,114	North (%) *N* = 1,263	Northeast (%) *N* = 7,700	Centre-west (%) *N* = 1,572	Southeast (%) *N* = 5,027	South (%) *N* = 2,552
Dentists' profile						
Graduate studies	52.8	43.9	48.3	52.2	57.4	62.4
Master's degree and/or doctoral studies	6.6	6.5	5.7	6.1	7.5	7.6
Tutor undergraduate students	9.7	6.0	8.8	5.2	12.5	11.8
Hiring by public tender test^∗^	50.0	42.8	41.6	57.7	46.9	79.8
Civil servant	44.1	40.9	40.1	55.7	37.7	63.4
Local government direct contract	82.1	90.4	86.4	79.4	72.3	85.9
Two years or less in this dental team	57.3	62.3	61.0	48.9	53.2	56.7
Career plan	20.1	21.4	11.8	23.6	21.4	39.7
Career plan with progression for time of service	16.5	16.8	9.1	19.8	17.3	34.8
Career plan with progression for professional performance	10.5	10.1	5.2	8.5	13.6	21.8
Career plan with progression for titles and professional improvement	15.7	17.7	9.2	20.5	15.0	32.2
Gain of financial incentive for good performance	22.9	15.9	23.9	20.1	22.5	26.1
Work management						
PHC unit a trainee field for undergraduate students	16.2	11.4	16.3	11.3	17.3	19.3
Documents proving the monthly planning and scheduling of activities	51.0	41.2	52.4	44.9	52.0	53.2
Monitoring and analysis of population's oral health indicators	66.4	53.4	67.8	55.9	71.6	64.8
Dental team receive support to plan and organise the labour process	79.0	67.1	80.2	71.7	83.0	77.7
Dental team have access to data to analyse population's oral health status	73.4	58.9	75.1	64.0	77.4	73.6
Documents proving self-evaluation in the past six months	55.9	29.0	60.4	40.5	62.4	52.3
Dental team take part in the PHC team meetings	66.3	57.2	68.0	66.2	63.3	72.1
Flexible dental appointment list (schedule and walk in patients)	90.0	84.7	89.0	88.0	92.4	92.4
Scheduling of dental appointments at any day and time	47.6	36.9	35.9	54.8	63.1	53.1
Referral for secondary care	75.0	59.0	71.8	63.5	83.3	83.7

^∗^Being approved in a public test that guarantee job stability.

**Table 3:** Mean, median, minimum, and maximum values of the quantitative independent variables related to dentists' profile and work management of PHC dental teams, Brazil, and according to each Brazilian Geographical Region, 2013-2014.

Dentists profile and management items		Brazil	North	Northeast	Centre-west	Southeast	South
Continuing professional development training^∗^	Mean	2.2	1.5	2.0	1.9	2.6	2.8
	Median	2.0	1.0	2.0	2.0	3.0	3.0
	Std. deviation	1.67	1.38	1.52	1.61	1.75	1.74
	Minimum	0	0	0	0	0	0
	Maximum	8	7	8	7	8	8

Number of PHC teams the dental team assist^∗∗^	Mean	1.2	1.1	1.2	1.2	1.4	1.3
	Median	1.0	1.0	1.0	1.0	1.0	1.0
	Std. deviation	0.67	0.40	0.57	0.59	0.82	0.75
	Minimum	1	1	1	1	1	1
	Maximum	9	8	9	5	9	9

^∗^Provided by the SUS; ^∗∗^one PHC team cover about 3,500 patients.

**Table 4:** Simple linear regression and adjusted model using dental team performance as dependent variable and dentists' profile and work management issues as independent variables. Brazil, 2013-2014.

Dentists profile and management items	Simple linear regression				Adjusted model					
	*B*	Std. error	*B* stand.	Sig.	*B*	Std. error	*B* stand.	Sig.	Confidence interval (95%)	VIF
Graduate studies	0.287	0.012	0.175	<0.001	0.151	0.011	0.092	<0.001	0.129–0.173	1.082
Tutor undergraduate students	0.479	0.020	0.174	<0.001	0.108	0.020	0.039	<0.001	0.069–0.147	1.213
Hiring by public tender test	0.179	0.012	0.109	<0.001	0.077	0.012	0.047	<0.001	0.054–0.101	1.241
Career plan with progression for time of service	0.378	0.016	0.172	<0.001	0.074	0.020	0.034	<0.001	0.035–0.113	1.899
Career plan with progression for professional performance	0.484	0.019	0.182	<0.001	0.128	0.024	0.048	<0.001	0.082–0.175	1.832
Financial incentive for good performance	0.328	0.014	0.169	<0.001	0.067	0.014	0.034	<0.001	0.040–0.094	1.162
Continuing professional development training (1-8 activities)^∗^	0.177	0.003	0.362	<0.001	0.101	0.004	0.206	<0.001	0.093–0.108	1.392
PHC unit a trainee field for undergraduate and graduate students	0.362	0.016	0.163	<0.001	0.058	0.016	0.026	<0.001	0.027–0.090	1.206
Documents proving the monthly planning and scheduling of activities	0.192	0.012	0.117	<0.001	0.044	0.011	0.027	<0.001	0.022–0.067	1.134
Monitoring and analysis of population's oral health indicators	0.392	0.013	0.227	<0.001	0.132	0.013	0.076	<0.001	0.106–0.158	1.336
Dental team receive support to plan and organise the labour process	0.376	0.015	0.188	<0.001	0.054	0.016	0.027	0.001	0.023–0.085	1.478
Dental team have access to data to analyse population's oral health status	0.391	0.013	0.211	<0.001	0.091	0.015	0.049	<0.001	0.061–0.120	1.568
Documents proving self-evaluation in the past six months	0.221	0.012	0.134	<0.001	0.070	0.012	0.042	<0.001	0.047–0.092	1.137
Dental team take part in the PHC team meetings	0.250	0.013	0.145	<0.001	0.040	0.012	0.023	0.001	0.017–0.063	1.100
Flexible dental appointment list (scheduled and walk in patients)	0.426	0.020	0.156	<0.001	0.281	0.018	0.103	<0.001	0.245–0.316	1.021
Scheduling of dental appointments at any day and time	0.190	0.012	0.116	<0.001	0.148	0.011	0.090	<0.001	0.126–0.169	1.011
Referral for secondary care	0.409	0.014	0.217	<0.001	0.174	0.013	0.092	<0.001	0.149–0.200	1.100
Number of PHC teams the dental team assist (1-9) ^∗^	0.064	0.009	0.053	<0.001	0.038	0.008	0.031	<0.001	0.023–0.054	1.005

^∗^Quantitative variables. Note: the variables “master's degree/doctoral studies,” “dentist's employer (local government direct contract),” “career plan,” “career plan with progression for titles and professional improvement,” “employment bond (civil servant),” and “time the dentist work in the dental team (Two years or less at the dental team) were excluded from the adjusted model.

**Table 5:** Beta values of the variables that showed statistical significance (*p* ≤ 0.05) in the adjusted multiple linear regression models, according to each Brazilian Geographical Region. 2013-2014.

Dentists profile and management items	Beta (CI 95%)				
	North (*n* = 1,263)	Northeast (*n* = 7,700)	Centre-west (*n* = 1,572)	Southeast (*n* = 5,027)	South (*n* = 2,552)
Graduate studies	0.107 (0.023–0.192)	0.133 (0.100–0.166)	0.146 (0.072–0.220)	0.140 (0.102–0.177)	0.124 (0.072–0.176)
Tutor undergraduate students		0.162 (0.098–0.226)			0.108 (0.023–0.193)
Hiring by public tender test				0.134 (0.095–0.174)	
Civil servant	0.199 (0.114–0.284)		0.101 (0.026–0.176)		
Local government direct contract		0.050 (0.002–0.098)			0.087 (0.017–0.158)
Career plan with progression for time of service				0.075 (0.005–0.144)	0.155 (0.100–0.210)
Career plan with progression for professional performance				0.124 (0.048–0.199)	
Financial incentive for good performance		0.124 (0.084–0.164)	0.176 (0.082–0.270)		0.174 (0.113–0.235)
Continuing professional development training (1-8 activities)^∗^	0.090 (0.059–0.122)	0.092 (0.080–0.105)	0.104 (0.080–0.128)	0.064 (0.052–0.076)	0.066 (0.050–0.083)
PHC unit a trainee field for undergraduate and graduate students		0.070 (0.022–0.119)		0.078 (0.027–0.129)	0.119 (0.048–0.190)
Documents proving the monthly planning and scheduling of activities				0.080 (0.041–0.118)	0.077 (0.027–0.127)
Monitoring and analysis of population's oral health indicators	0.195 (0.107–0.283)	0.127 (0.086–0.168)	0.145 (0.070–0.219)	0.164 (0.119–0.210)	0.097 (0.039–0.155)
Dental team receive support to plan and organise the labour process	0.165 (0.068–0.262)	0.059 (0.009–0.109)			
Dental team have access to data to analyse population's oral health status		0.118 (0.071–0.165)		0.106 (0.056–0.156)	0.150 (0.087–0.214)
Documents proving self-evaluation in the past six months	0.096 (0.05–0.187)	0.080 (0.046–0.114)	0.094 (0.020–0.167)	0.059 (0.020–0.099)	
Dental team take part in the PHC team meetings		0.063 (0.026–0.099)	0.108 (0.031–0.186)	0.050 (0.011–0.089)	0.102 (0.044–0.160)
Flexible dental appointment list (scheduled and walk in patients)	0.232 (0.118–0.347)	0.298 (0.245–0.351)	0.231 (0.122–0.340)	0.156 (0.088–0.224)	0.240 (0.147–0.333)
Scheduling of dental appointments at any day and time	0.100 (0.016–0.185)			0.091 (0.053–0.128)	0.106 (0.057–0.155)
Referral for secondary care		0.095 (0.057–0.133)	0.191 (0.115–0.268)	0.214 (0.163–0.246)	0.126 (0.058–0.194)
Number of PHC teams the dental team assist (1-9)^∗^	0.117 (0.015–0.218)				

^∗^Quantitative variables. Note: the table presents only the variables that showed statistical significance (*p* ≤ 0.05) in the adjusted multiple linear regression model of each Brazilian Geographical Region. The variables “master's degree/doctoral studies,” “career plan,” “career plan with progression for titles and professional improvement,” and “time the dentist works in the dental team (two years or less at the dental team)” were excluded from the adjusted model of all regions and are not presented in the table above.

## Data Availability

The data used to support the findings of this study are available from the corresponding author upon request.
